# Editorial: The contribution of autophagy to neuronal metabolism

**DOI:** 10.3389/fcell.2025.1604307

**Published:** 2025-04-25

**Authors:** Francois Singh, Genta Ito, Fumi Suomi

**Affiliations:** ^1^ Biomedical Center, Faculty of Medicine, School of Health Sciences, University of Iceland, Reykjavik, Iceland; ^2^ Department of Biomolecular Chemistry, Faculty of Pharmaceutical Sciences, Teikyo University, Tokyo, Japan; ^3^ University of Helsinki, Stem Cells and Metabolism Program, Faculty of Medicine, Helsinki, Finland; ^4^ HiLife, University of Helsinki, Helsinki, Finland

**Keywords:** autophagy, neurodegenerative diseases, mitophagy, metabolism, neurons

In 1963, Christian de Duve first postulated the concept of “autophagy” (de Duve, 1963). The term has since then been universally adopted to describe the evolutionary conserved mechanism that allows the degradation and recycling of intracellular components. De Duve was awarded the Nobel prize in Physiology and Medicine in 1974, and Yoshinori Ohsumi’s work on autophagy led to him being awarded the Nobel prize in 2016 (Harnett et al., 2017). In the past years, our understanding of the different pathways involved in autophagy has sharply increased. An alteration of this process has been shown to play a pivotal role in the aetiology of multiple pathologies, including neurodegenerative diseases, cancer, metabolic disorders, and immunological diseases (Kumar et al., 2021). Neurodegenerative diseases are a heterogenous group of currently uncurable diseases. As age is the biggest risk factor for neurodegenerative diseases, the number of patients affected will inevitably rise with the ageing of the population. Recently, both autophagy and mitophagy were profiled in the ageing brain of mice, highlighting an age-related increase of mitophagy in multiple brain neuronal and glial populations (Rappe et al., 2024). Neurons, with their complex architecture and post-mitotic nature, require efficient mechanisms to recycle damaged components and sustain metabolic demands (Suomi and McWilliams, 2019). Despite their longevity, neuronal proteins and organelles have a shorter lifespan, necessitating continuous turnover. Recent studies have highlighted the critical role of neural macroautophagy and the dynamic metabolic response in both health and disease, with knockout models in mice demonstrating severe neurodegenerative and developmental defects (Hara et al., 2006; Komatsu et al., 2006; Suomi and McWilliams, 2019). In humans, mutations in autophagy-related genes (ATG) have been linked to various neurological disorders, including static encephalopathy (Collier et al., 2021; Saitsu et al., 2013) and congenital ataxia (Kim et al., 2016). Autophagy-deficient cancer cells are reported to switch to glycolytic metabolism maintain cell growth (Feng et al., 2018). Interestingly, similar metabolic switch to glycolysis is reported to be the driver of the sporadic AD patient-derived neurons (Traxler et al., 2022). However, the specific contributions of autophagy, particularly selective autophagy like mitophagy, to nervous system metabolism remain underexplored, necessitating further investigation.

This Research Topic includes five articles: one original research article by Motomura et al., three reviews by Green et al., Mohammed et al., Sakurai and Kuwahara, as well as a mini review article by Fernandes et al..

In their review article, Sakurai and Kuwahara provide an overview of the current understanding of the contribution of canonical and non-canonical autophagy in Parkinson’s disease (PD). PD is the second most common neurodegenerative disorder (Coppedè, 2012), and the number of patients with PD is estimated to reach 1.5 billion by 2050 (United Nations - Department of Economic and Social Affairs, 2019). The authors describe the involvement of macroautophagy, microautophagy, and chaperone-mediated autophagy, as well as recent findings on the potential role of conjugation of ATG8 to single membranes (CASM) in PD (Durgan and Florey, 2022; Figueras-Novoa et al., 2024). The review highlights the need to better understand the different roles of autophagy in understanding the aetiology of PD and emphasises the limitations of the current cell and animal models used in PD research. An improvement in both domains could lead to novel curative strategies for PD.

In their mini review article, Fernandes et al. provide a comprehensive overview of the potential of modulating autophagy as a therapeutic strategy for clearing aggregated proteins and contributing to the metabolites balancing in Alzheimer’s disease (AD). The mini-review summarises the different components of the autophagy machinery that can be affected in AD, and discusses the limitations and challenges of using autophagy modulators in clinical studies in the context of AD treatment. The review highlights that while autophagy modulation could hold promise for clearing aggregated proteins, challenges remain into finding autophagy modulators with higher specificity and biomarkers that surrogate autophagy activity in the human brain.

The involvement of autophagy in diseases goes far beyond neurodegenerative diseases and the review by Mohammed et al. provides an extensive review of the role of autophagy in the onset and progression of hepatocellular carcinoma (HCC). The authors highlight the dual nature of autophagy in both suppressing and promoting HCC depending on the context. In the early stages of tumorigenesis, autophagy prevents the initiation, proliferation, and metastasis of tumours whereas more advanced tumours, autophagy increases the resistance of tumour cells to drugs. This review hence highlights the challenges of targeting autophagy in the treatment of HCC.

Mitophagy is the targeted degradation of mitochondria by the autophagy machinery. This process is used by the cells during metabolic remodelling and to remove dysfunctional or damaged mitochondria, which can occur during the normal functioning of cells, as well as in pathological contexts. Altered mitophagy has been linked to neurodegenerative diseases (Antico et al., 2025; Burté et al., 2015; Singh et al., 2021; Singh et al., 2024; Singh and Ganley, 2021). Hence, targeting mitophagy as a therapeutic solution has become increasingly attractive in recent years, with the development of USP30 inhibitors, PINK1 activators, and LRRK2 inhibitors (Antico et al., 2025; Liu et al., 2022; Singh et al., 2021; Tasegian et al., 2021). Fusion and fission of the mitochondrial network as well as mitochondrial motility are essential to maintain mitochondrial homeostasis. In their review, Green et al. provide an extensive overview of the molecular mechanisms that control these dynamic events as well as potential pharmacological interventions for mitochondrial disorders.

In their original research article, Motomura et al. investigate the effect of novel aromatic turmerone (Ar-turmerone) analogue capable of activating nuclear factor erythroid 2-related factor 2 (Nrf2) and chaperone-mediated autophagy (CMA). Nrf2 has been recognised as a master regulator of redox homeostasis, mitochondrial biogenesis, metabolism, and proteostasis, with an implication in a variety of pathological conditions (Ljubicic et al., 2010; Zhang, 2025). In their article, the authors focus on the effects of four Ar-turmerone analogs on CMA in an *in vitro* model using SH-SY5Y cells. Interestingly, the authors find that A4 is capable of activating CMA in an Nrf2-independent manner, through the phosphorylation of p38. This activation of CMA is able to protect cells against rotenone, a mitochondrial toxicant that inhibits complex I of the mitochondrial electron transport chain. This article highlights the development of new creative and promising therapeutic approaches to prevent neurodegenerative diseases by activating CMA.

In summary, the articles in this Research Topic highlight the complex nature of modulating autophagy and mitochondrial homeostasis in pathological contexts such as neurodegenerative diseases and hepatocellular cancer. Significant advances have been made in the recent years, but further understanding of the molecular pathways associated with autophagy will lead to refinement of therapeutic approaches.

We would like to thank all the authors and referees that have contributed to this Research Topic, as well as the Editorial Office at Frontiers without whom this special Research Topic would not have been possible.
